# Assessment of factors influencing oral health-related quality of life (OHRQoL) of patients with removable dental prosthesis

**DOI:** 10.12669/pjms.36.2.1257

**Published:** 2020

**Authors:** Modhi Al Deeb, Tariq Abduljabbar, Fahim Vohra, Muhammad S. Zafar, Mudassir Hussain

**Affiliations:** 1 Modhi Al Deeb, Department of Prosthetic Dental Science, College of Dentistry, King Saud University, Riyadh 11545, Saudi Arabia; 2 Tariq Abduljabbar, Department of Prosthetic Dental Science, Research Chair for Biological Research in Dental Health, College of Dentistry, King Saud University, Riyadh 11545, Saudi Arabia; 3 Fahim Vohra, Department of Prosthetic Dental Sciences, College of Dentistry, King Saud University, Riyadh 11545, Saudi Arabia; 4 Muhammad S. Zafar, Dept. of Restorative Dentistry, College of Dentistry, Taibah University, Al Madina Al Munawara, 41311, Saudi Arabia; 5 Mudassir Hussain, Department of Community and Preventive Dentistry, Karachi Medical and Dental College, Karachi, Pakistan

**Keywords:** Oral health Quality of Life, Bridge, Crown, Dentistry, Denture, Prosthodontics

## Abstract

**Objective:**

The aim of this study was to evaluate factors affecting oral health related quality of life (OHRQoL) of patients using removable dental prosthesis.

**Methods:**

The present study employed a cross sectional analytical design. A total of 200 patients participated and interviewed using a cross sectional analytical design. In the first section of the questionnaire patients were asked about demographic data whereas the second part of the questionnaire assessed medical history, oral habits, smoking status, oral hygiene habits and frequency of dental visit. The questionnaire also collected information regarding patient’s removable prosthesis. Questionnaire of OHIP-DENT (Oral Health Impact Profile) was also employed to measure oral health quality of life (OHRQoL) on the domains of functional limitation (FL), physical pain (P1), psychological discomfort (P2), physical disability (D1), psychological disability (D2), social disability (D3) and handicap (H). Relationships between the demographic, socio-economic and education variables and others OHIP-EDENT scores were explored by comparing mean scores by applying ANOVA.

**Results:**

The study participants comprised of 107 males (53.5%) and 93 females (46.5%). Regarding oral care, participants reporting to visit their dentist within one year were 40.0%. The highest score was recorded for the functional limitation (FL) domain (15.62±6.6), followed by social disability (D3) (15.23±5.06) and physical pain (P1) (14.28±4.8). The respective scores for physical (D1) and psychological disability (D2) and handicap (H) were 10.47±4.84, 11.32±5.38 and 12.45±4.50 respectively.

**Conclusions:**

Removable partial denture patients showed minimum problems with mastication, social compromise and functional discomfort. The oral health quality of life of removable denture patients is significantly influenced by patient education level, socio-economic status, medical conditions, smoking and tobacco use habits.

## INTRODUCTION

The term “quality of life” was coined decades ago and has gained popularity with time.^1^ The quality of life (QoL) is evidently influenced by the individual’s ability to participate in daily life activities that in turn may be affected by factors such as social circle, economic status and stress.^2^ Based on the fact that the oral health is associated with socio-economic and psychological aspects, it is considered as an important component of quality of life assessment. The World Health Organization (WHO) has recognized oral health related quality of life (OHRQoL) an integral part of global oral health program.^3^

In recent years, the remarkable advancement in the quality of life lead to improvement of living standard, access to healthcare and average life. In terms of oral health for instance, relatively more number of remaining teeth are observed in the aging population.^4^ However, still there are growing population suffering from loss of multiple teeth and the need for removable or fixed dental prosthesis.^5^

Teeth are vital component of personality required for speech, mastication and aesthetic for everyday routine life. Having dental prosthesis instead of natural teeth may deteriorate patient’s quality of life in a number of ways such as possible functional or aesthetics compromises, lack of retention or stability and psychological impact.^6^ In order to diminish such effects, it is important to assess the factors affecting the oral health related quality of life. Therefore, the OHRQoL assessment has become a vital tool for patient oriented dental research. A number of indices and research tools have been used for this purpose such as Geriatric Oral Health Assessment Index^7^ Oral Health Impact Profile (OHIP)^8^ and various multiple-choice questionnaires. The OHIP is a validated research tool for assessing the oral health related quality of life^9^ and has been used in this study to assess the response of subpopulation.^10^ It is hypothesized that factors effecting OHQoL factors for Removable partial denture patients can be identified as an outcome of the study. The aim of this study was to evaluate various factors affecting the oral health related quality of life of patients using removable dental prosthesis.

## METHODS

### Study Design

The study designed, conducted and reported following the Consolidation Standards of Reporting Trials (CONSORT) Statement. The present study was performed following guidelines recognized by the Declaration of Helsinki as revised in 2013 for experimentation involving human patients.(FR-0578 December 14, 2018) All participants were informed that they could withdraw their participation at any stage of the investigation without consequences. This study was conducted from May 2018 to May 2019 after board review at a specialist dental center in Riyadh city, Saudi Arabia, using a cross sectional analytical design.

### Participant

Patients were recruited from specialist dental practice for prosthodontics in Riyadh, Saudi Arabia. A sample of 200 participants was considered adequate based on calculations from previous studies.^4^,^5^ Patients having removable prosthodontic treatment for replacement of missing or lost teeth by prosthodontic specialists were included. A total of 200 patients consented to participate and were interviewed by telephone to identify the patient initially, to confirm previous treatment and their selection according to inclusion and exclusion criteria.

Medically healthy patients who had removable prosthodontic treatment in the last two years were included. Patients with life threatening conditions, physical and psychological ailments, those treated by non-specialist and patients with history of malignancy, chemotherapy or radiotherapy in head and neck region were excluded. Patients were assured that all information was strictly confidential.

### Questionnaire and data collection

The questionnaires were available in English and Arabic languages. The questionnaire used in this study comprised of two sections; first section assessed the demographic data of the participants including name, age, ethnicity, gender, education [Primary (up to standard five), Secondary (up to standard nine) and tertiary education]; marital and socio-economic status. The second section assessed the medical history, oral habits, smoking status, oral hygiene habits and frequency of dental visit. The questionnaire also collected information regarding prosthesis such as the number of teeth, type of prosthesis (complete or partial), location of prosthesis, duration of function and number of prosthesis. All patients were evaluated at a review appointment and requested to complete the questionnaires.

A second questionnaire of OHIP-DENT (Oral Health Impact Profile)^10^ was employed to measure oral health quality of life (OHRQoL). The main domains assessed in the OHIP-DENT questionnaire were functional limitation (FL), physical pain (P1), psychological discomfort (P2), physical disability (D1), psychological disability (D2), social disability (D3) and handicap (H). The OHIP-DENT questionnaire comprised of 19 items giving the choice of responding in five categories for each item:

1) Never
2) Hardly ever
3) Occasionally
4) Fairly often
5) Very often.

In addition, patients were also asked regarding their satisfaction from the prosthesis. Patients responded according to a Likert scale:

1) Totally satisfied
2) Very satisfied
3) Reasonably satisfied
4) Not very satisfied
5) Not at all satisfied.^11^

All responses to questions were coded and entered into a spreadsheet by a single operator.

### Statistical analysis

The data analysis and recordings were carried out using the Statistical Package for Social Sciences (SPSS) version 21.0. The means and standard deviations for the mean scores for the overall participants was identified using descriptive statistics. The frequency distributions of all responses were computed. Relationships between the demographic, socio-economic and education variables and others OHIP-EDENT scores were explored by comparing mean scores by applying ANOVA.

## RESULTS

### Characteristics of Participants

A total of 200 subjects were included in the study according to the inclusion criteria. The general characteristics of participants such as gender, education level, marital status, socioeconomic status, smoking, habits and medical conditions are shown in Table-I. The study participants comprised of 107 males (53.5%) and 93 females (46.5%); while the majority (154, 80.6%) of participants were married, and 37(19.4%) were unmarried. In terms of education, 34.5% were illiterate; approximately 21% went to school and nearly 23.5% attended college. Only 15.5% of the subjects attended university. Most of participants (92.5%) belonged to low or middle socioeconomic status, while only 4.5% were from high class. Most of participants (69%) reported no habit of using tobacco or gutka, while tobacco and pan/gutka was being used by 12.5% and 13.5% of participants’ respectively. 66.5% were never-smokers, 11% were previous smokers and 22.5% reported to be smoking currently. In terms of medical conditions, 50.5% reported no significant medical conditions while 49.5% had some kind of systemic illness such as cardiovascular (11%), diabetes (23%), arthritis, GIT & dryness of mouth (7.5%), hepatitis, HIV & muscular disorder (7%).

**Table-I:** Distribution of Socio-Demographic variables, habits & medical conditions.

	Variables	No (%)
Gender	Male	107(53.5)
	Female	93(46.5)
Marital Status	Unmarried	39(19.5)
	Married	161(80.5)
Education Level	Illiterate	69(34.5)
	School	42(21.0)
	College	47(23.5)
	University	31(15.5)
	No response	11(5.5)
Socioeconomic Status	Low level	90(45)
	Middle level	95(47.5)
	High level	9(4.5)
	No response	6(3.0)
Habits	Tobacco related	25(12.5)
	Pan, Ghutka and others	27(13.5)
	Ghutka and others	10(5.0)
	None	138(69)
Smoking	Smoker	45(22.5)
	Past smoker	22(11)
	Non-smoker	133(66.5)
Medical conditions	Cardiovascular	22(11)
	Diabetes	46(23)
	Arthritis, GIT & Dryness of mouth	17(8.5)
	Hepatitis, HIV & muscular disorder	14(7)
	Nothing significant	101(50.5)

### Oral and prosthodontic care of Participants

The participant’s interest for dental care was assessed recording dental visit frequency and oral hygiene habits (Table-II). Percentage of participants reported to visit their dentist within one year, 2-5 years and after five years or later was 40.0%, 29.5% and 30.6% respectively. Equal numbers of participants were brushing their teeth once or twice a day (32.5%) while the remaining 35% were not regular in brushing their teeth. The participants of this study were using a variety of dental prostheses (Table-II); including complete dentures (CD) in lower (L) jaw (2.0%), upper (U) jaw (2.5%), CD in both U/L jaw (26.5%), removable partial denture (RPD) with CD (8.5%), crowns (7.8%), fixed partial dentures (FPD) (12.0%), RPD in L jaw (7.5%), RPD in U jaw (8.5%) and RPD in both jaws (18.5%).

**Table-II:** Distribution of Dental care of study subjects.

	Variables	No (%)
Time period of Dentist visit	<1 year	80(40.0)
	2 to 5 years	59(29.5)
	> 5 years	61(30.6)
Frequency of Brushing	1 time in a day	65(32.5)
	2 times in a day	65(32.5)
	Not regular	70(35.0)
Type of Prosthesis use	CD:L	4(2.0)
	CD:U	5(2.5)
	CD:U/L	53(26.5)
	RPD/CD	17(8.5)
	RPD:L	15(7.5)
	RPD:U	17(8.5)
	RPD:U/L	37(18.5)

### Measurement of Domains of OHIP-EDENT

The OHIP-DENT score of participants was calculated for each domain for their mean and standard deviation (SD) values (Table-III). The highest score was recorded for the functional limitation (FL) domain (15.62±6.6), followed by social disability (D3) (15.23±5.06) and physical pain (P1) (14.28±4.8). The respective scores for physical disability (D1), psychological disability (D2) and handicap (H) were 10.47±4.84, 11.32±5.38 and 12.45±4.50 respectively. The lowest score was recorded for psychological discomfort (P2) (7.61±4.11) Table-III.

**Table-III:** Descriptive statistics of Scores of different Domains of OHIP-EDENT.

Domains	Mean (SD)
Functional Limitation (FL)	15.62(6.6)
Physical pain (P1)	14.28(4.8)
Psychological discomfort (P2)	7.61(4.11)
Physical disability (D1)	10.47(4.84)
Psychological disability(D2)	11.32(5.38)
Social disability (D3)	15.23(5.06)
Handicap (H)	12.45(4.50)

**Fig.1:**
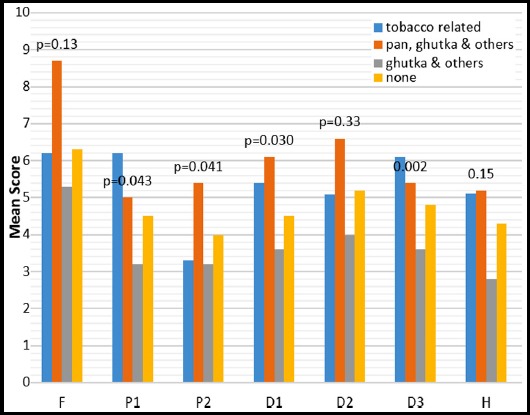
Realtionship of differnet dimension of OHIP-DENT with habits of subjects. F: Functional limitation, P1: Physical pain, P2: Psychological discomfort, D1: Physical disability, D2: Psychological disability, D3: Social disability, H: Handicap

**Fig.2:**
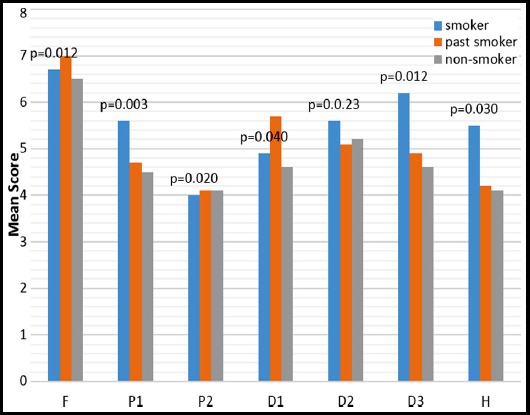
Relationship of different dimension of OHIP-DENT with smoking status of subjects. F: Functional limitation, P1: Physical pain, P2: Psychological discomfort, D1: Physical disability, D2: Psychological disability, D3: Social disability, H: Handicap

Males recorded higher impact in all domains except *psychological discomfort* and *handicap*. A significant relationship was found between gender and *psychological* (p=0.020) *and social disability* (p=0.040), with the males scoring higher than females. Patients who had no formal schooling or schooling up to standard 5 reported much lower impacts than patients who had secondary or tertiary education. A significant relationship was found between education and *functional limitation* (p=0.030) and *physical disability* (p=0.040). Patients who were in a higher income group generally reported more OHRQoL impacts than patients who earned a lower salary. Patients who had no source of income recorded the lowest impacts for *social disability* (Mean=5.4, p=0.360) and *handicap* (Mean=6.2, p=261). A significant relationship was found between economic status and psychological disability (p=0.01) and psychological discomfort (p=0.03). Patients with no medical condition reported higher impact scores in all domains. Significant relationships were also found between the physical pain and psychosocial domains with general medical health. Patients who were habitual users of pan, ghutka & others, generally reported more OHIP-DENT impacts than patients who were not involved in any habits (Fig.1). Significant relationships were found in physical and psychosocial domains. Patients who were smokers reported higher impact scores in all OHIP-DENT domains (Fig.2). Significant relationships were found between functional limitation, physical and psychosocial domains.

## DISCUSSION

This study aimed at assessing the various factors influencing oral health-related quality of life (OHRQoL) of patients with removable dental prosthesis. The tool (OHIP) used in this study measured various dimensions of OHRQoL i.e. Functional Limitation (FL), Physical pain (P1), Psychological discomfort (P2), Physical disability (D1), Psychological disability (D2), Social disability (D3), Handicap (H). All these domains were compared with various influencing factors such as Education Level, Socioeconomic Status, Habits, Smoking and Medical condition of the patients. The sample consisted 200 patients with an almost equal proportion of male and female participants. However, there was a lot of variation in the other factors influencing OHRQoL, as shown in the results.

In this study, the highest OHIP mean values were observed in domains of functional limitation, and social disability. Suggesting that most of participant’s difficulties were related to mastication, speech, bad odor, taste and type of food, in addition to communication, social interactions, ill-fitting denture and inadequate retention.^12^,^13^ This signifies that participants persevered with great functional difficulty as it was not painful. Most edentulous patients feel helpless and believe that they have to accept denture problems as part of wearing a prosthesis.^14^ On the other hand, the OHIP mean scores were the lowest in psychological discomfort. These results are comparable to a recent study by Kranjcic J et al.^15^ according to which, younger participants, members from rural places, those with lower levels of education, and shorter period of denture wearing demonstrated a higher impact on OHRQoL. Kranjcic J et al.^15^ showed that OHRQoL was significantly affected by the participants’ age, education, profession, residence place size, type of prosthesis, and the time of denture wearing period. In the present study, significant differences were found between the various domains of OHIP and the patients’ gender, education status, general health condition of the patients and their smoking status. Whereas no significant differences could be found with socio-economic status, habits of the patients and the type of their prosthesis.

Studies have shown that factors such as age, education, profession, type of prosthesis and the time of denture wearing period significantly affect OHRQoL.^16^,^17^ However, in our sample of patients, gender and psychological discomfort showed a significant relationship (p=0.040) with the males, scoring higher than females. Similar results were reported in a previous OHRQoL study on fixed and removable partial dental prostheses.^18^ However, age was more significantly associated with OHRQoL than gender in such studies.^19^ In the present study, the educational status showed a statistically significant relationship with functional limitation (p=0.030) and physical disability (p=0.040) domains of OHRQoL. These results are in agreement with the results demonstrated in a comparable study by Dable et al.^19^ It is logical to assume that participants with lower level of education have greater expectations (even unrealistic) due to the lack of understanding of functional limitations of prosthodontic treatment, often comparing the dentures to their natural teeth.^20^

The study showed a significant relationship between the psychosocial domains and general health of the patients in diseases like Diabetes Mellitus, Cardiovascular diseases, Arthritis, Hepatitis and HIV AIDS. These results are in agreement with previous studies comparing the general health conditions with oral health of edentulous patients.^21^ Patients with chronic illness, undergo multiple extractions of teeth in addition to residual ridge resorption. If bone loss is progressive, it often leads to a clinical situation with insufficient bone support compromising prosthesis stability and retention.^21^,^22^ In addition, smoking status of patients assessed showed a statistically significant relationship with the OHRQoL in functional limitation, physical and psychosocial domains. These results conform to the results reported by Kotzer et al.^16^ according to which, patients who reported one or more impacts ‘fairly often’ or ‘very often’ were more likely to smoke daily, have oral pain, perceive their general health, mouth health and quality of life to be fair or poor and are dissatisfied with their teeth or dentures.

With regards to clinical implications of the findings, it is the authors opinion that patient’s expectations must be taken into consideration and all possible limitations must be discussed with them before embarking on an extensive removable prosthodontic treatment.^23^ A possible limitation of OHQoL studies are the subjective responses of patients which are influenced by patient behavioral and psychological attitude. Clinicians should also recognize the important role they play in improving patient’s quality of life by assessing factors like age, education, social status, type of prosthesis, habits and medical conditions of patients. Therefore, for a successful prosthodontic treatment outcome of removable denture patients, factors effecting oral health and quality of life including smoking, tobacco chewing habits, medical conditions, education and patient motivation must be addressed.

## CONCLUSION

Removable partial denture patients showed minimum problems with mastication, social compromise and functional discomfort. The oral health quality of life of removable denture patients is significantly influenced by patient education level, socio-economic status, medical conditions, smoking and tobacco use habits.

### Authors’ Contribution:

**FV:** Data collection, study design, manuscript writing, final manuscript approval, is responsible for integrity of research.

**MN:** Data collection, study design, manuscript drafting, data analysis, manuscript approval.

**MSZ and MH:** Data collection, manuscript approval and data interpretation.

**HRB:** Data collection, writing, revise, editing and final manuscript approval.
